# Clinical Significance of Molecular Diagnostic Tools for Bacterial Bloodstream Infections: A Systematic Review

**DOI:** 10.1155/2016/6412085

**Published:** 2016-11-16

**Authors:** Jean Pierre Rutanga, Therese Nyirahabimana

**Affiliations:** School of Science, College of Science and Technology, University of Rwanda, BP 56, Huye, Rwanda

## Abstract

Bacterial bloodstream infection (bBSI) represents any form of invasiveness of the blood circulatory system caused by bacteria and can lead to death among critically ill patients. Thus, there is a need for rapid and accurate diagnosis and treatment of patients with septicemia. So far, different molecular diagnostic tools have been developed. The majority of these tools focus on amplification based techniques such as polymerase chain reaction (PCR) which allows the detection of nucleic acids (both DNA and small RNAs) that are specific to bacterial species and sequencing or nucleic acid hybridization that allows the detection of bacteria in order to reduce delay of appropriate antibiotic therapy. However, there is still a need to improve sensitivity of most molecular techniques to enhance their accuracy and allow exact and on time antibiotic therapy treatment. In this regard, we conducted a systematic review of the existing studies conducted in molecular diagnosis of bBSIs, with the main aim of reporting on clinical significance and benefits of molecular diagnosis to patients. We searched both Google Scholar and PubMed. In total, eighteen reviewed papers indicate that shift from conventional diagnostic methods to molecular tools is needed and would lead to accurate diagnosis and treatment of bBSI.

## 1. Introduction

Bloodstream infection (BSI) is a life-threatening condition caused by the presence of microorganisms, generally bacteria or fungi, in the blood [1]. Bacterial bloodstream infection (bBSI), caused by a range of bacteria, can be distinguished as either community acquired or hospital acquired and lead to high morbidity and mortality rates all over the world [2]. Bacterial virulence factors gain access to the blood circulation and are thereafter presumed to cause target organ damage [3]. Culture-based techniques are still of considerable interest for the detection and identification of pathogens causing bBSI.

The presence of bacteria and bacterial products in circulating blood has been known for decades. Thus, detection and identification of bacteria based on detection of circulating nucleic acids has been a constant and ongoing challenge [4]. Polymerase chain reactions (PCR) assays which can be done on blood collected in an anticoagulant (EDTA) tube are highly promising. In the absence of specific clinical certainty, broad-range PCR, using primers targeting the 16S rRNA gene, the 23S rRNA gene, and the rpoB gene, are particularly suitable as they are ubiquitous to all bacteria [5]. In addition, matrix-assisted laser desorption/ionization time-of-flight mass spectrometry (MALDI-TOF MS) is highly utilized for high-throughput identification of bacteria from agar plates [2].

The current gold standard method of bloodstream microbial detection and identification is the blood culture (BC). The latter is currently based on an automatic and continuous manipulation of liquid culture, followed by gram staining, subculture, and the use of phenotypic methods to identify the bacteria and their associated antibiotic susceptibility. A major disadvantage to culture is the time required to complete the entire described process, which normally ranges from 1 to 5 days or more [6]. Results from traditional BC are usually not available before 24 to 72 hours after the initial patient presentation to the clinic. In resource poor healthcare settings, as BC runs slowly, this can sometimes oblige the physicians to prescribe nonspecific antibiotic treatment to patients necessitating initial use of empirical therapy [7, 8]. Therefore, a quick detection of bacterial infection is one of the most crucial and foreseen functions of most of laboratory bacteriology units. Therefore, the discovery and application of rapid and reliable diagnostics for bBSI would represent a major unattainable need in curing seriously ill patients [4]. In this regard, we conducted a systematic review on currently available molecular diagnostics for bBSI with the main aim of presenting their clinical significance in healthcare settings.

## 2. Methods

### 2.1. Data Source

Published papers related to our review topic were carefully searched through Google Scholar and PubMed searching tools. Google Scholar has been of our interest because it covers a broad range of scientific papers in different research areas; the same is for PubMed which covers around 26 million of biomedical papers from Medline and life science journals. Both databases are regularly updated with newly published papers.

### 2.2. Searching Strategy

The online paper search was conducted on two different dates (February 12 and 27, 2016). Our research was developed based on different searching keywords related to our review topic. We used the following keywords: “bacterial bloodstream infections”, “bBSIs”, “molecular diagnosis of bBSI”, and “clinical significance bBSI”. These three searching keywords or terms were entered in Google Scholar and PubMed. Thus, we considered and reviewed all papers published on clinical significance of molecular diagnostic tools for bBSI.

### 2.3. Inclusion and Exclusion Criteria

The papers were examined, extracted, and considered based on different inclusion and exclusion criteria we set. All these criteria were applied to paper titles we got for our first time of searching; therefore all chosen papers for our review have met and satisfied the following criteria: being “published in English,” “providing information on development of molecular diagnostic tools for bBSI and their clinical significance,” or being “published in 2000 to 2016.” However, other retrieved papers were rejected based on the following exclusion criteria: being “published before 2000,” “published in a language other than English,” or “published as a book.” After examining all these aforementioned criteria, papers were considered for review if they were available in full text through PubMed.

## 3. Results and Discussion

### 3.1. Results

**Figure 1:**
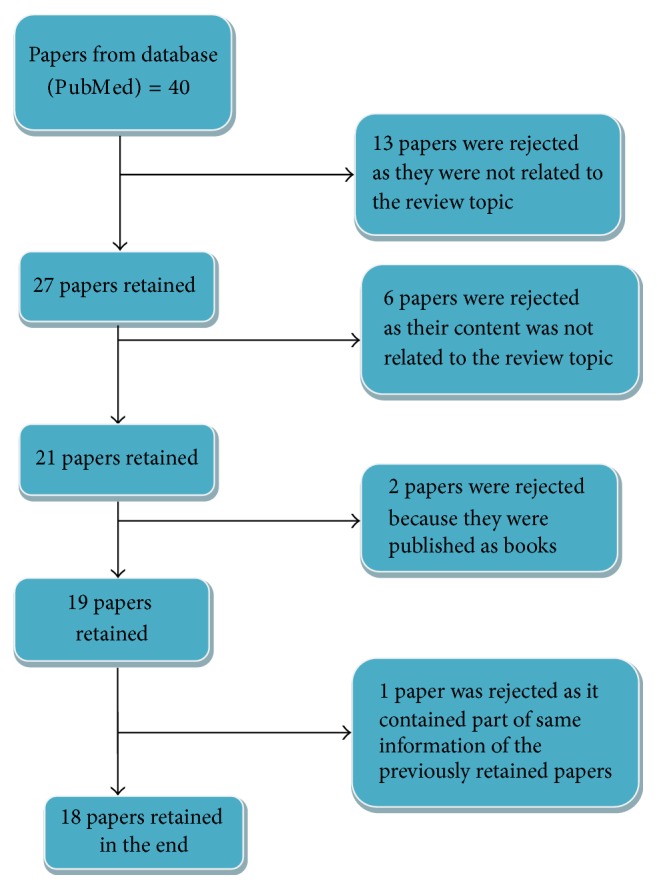
Flow diagram of paper selection.

A complete description of our review strategy is found on the flowchart presented in Figure 1. We retrieved 40 published papers which yielded 18 papers included in this review. Analysis of the total 18 papers included in this review was done based on the following aspects: year of publication, research country, sample size, patients' setting, type of diagnostic tool, research design, performance, and findings of the paper (Table 1).

The first category of the reviewed papers underlined the development and benefits of PCR-based assays. PCR-based methods have been discussed by 8 of the 18 reviewed papers. Dark et al., 2011, reported the usefulness of PCR techniques mostly by using universal probes, followed by sequencing, and highlighted their high sensitivity and specificity [11]. Liesenfeld et al., 2014, added that the PCR technique seems to be superior to BC given its accuracy of detecting bacteria and fungi [12]. Tennant et al., 2015, investigated the development of quantitative PCR (q-PCR) and its application in detecting* Salmonella *species [13]. Jordana-Lluch et al., 2015, revealed the sensitivity improvement and clinical accuracy of PCR [14]. Carrara et al., 2013, reported that the diagnosis challenges of BSIs could be decreased by use of PCR method, especially multiplex PCR, which can improve patient lives [15]. Lecuit and Eloit, 2014, advised that blood culture has to be supplemented with nucleic acid-based tests and PCR [16]. Lehmann et al., 2008, added that PCR-based diagnostic techniques are more accurate in terms of their sensitivity and specificity towards detection of target pathogen [17]. Chang et al. discussed the available molecular techniques by emphasizing more real-time PCR which is accurate and quick in detecting infection, thus reducing mortality and morbidity [18].

The second category of the reviewed papers compared the diagnostic significance of BC to PCR. Some reviewed publications reported on BC as a good technique in diagnosis of bBSI but also presented its various disadvantages such as long turnaround time, easy contamination, and false negative and positive results [8, 13, 15–17, 19]. A total of 12 out of 18 reviewed papers explored the BC's performance and recommended different and improved molecular techniques to detect BSIs with a particular emphasis on bacteria. Dark et al., 2011, mentioned the interest in the use of BC but also notified that PCR technology is more crucial as it enables detecting even minute organisms by using short turnaround time with emphasis on bacteria [11]. Jordana-Lluch et al., 2014, confirmed the limitations of BC, mainly centered in biochemical identification, and recommended a need for BC to be replaced by molecular techniques such as PCR [20]. This was also emphasized by Jordana-Lluch and his coauthors who reported that BC is the gold standard diagnostic tool for bBSI, but as it suffers from low sensitivity, it must be supplemented with no cultivable methods such as PCR [20]. Chang et al., 2013, underscored some pitfalls of BC such as a need for a long turnaround time and risk for contamination and concluded that affording molecular techniques, especially real-time multiplex PCR, would improve diagnosis of bBSI [18]. In the same line of overcoming BC's disadvantages, Chang with his colleagues proposed molecular tools other than conventional PCR such as DNA microarrays, RNA-based fluorescence, in situ hybridization probes, and real-time PCR [18].

The same comparison of BC to PCR was illustrated through different field trials. Most of the works by Faria et al., 2015, evaluated the Illumina sequencing of PCR amplified 16S rDNA samples collected from intensive care unit (ICU). As part of their findings, they suggested that a molecular approach may enable improved detection of polymicrobial infections. The application of sensitive molecular methods to clinical samples can identify more organisms in samples when compared to BC clinical diagnostics, which is selective for specific organisms. By working on patients' samples from ICU using real-time PCR, Dark et al., 2011, revealed a high diagnostic specificity and a 3- to 10-fold higher sensitivity for real-time PCR compared to conventional BC [11]. Jordana-Lluch et al., 2015, compared sensitivity, specificity, and positive and negative predictive values of blood culture to the ones of PCR coupled with electrospray ionization mass spectrometry (PCR/ESI-MS) evaluated on clinical samples and concluded that molecular techniques are performing far better than BC [14]. Wallet et al., 2010, by examining the ICU patients' samples using both BC and LightCycler-SeptiFast (LC-SF), obtained the following results: the positivity rate of BCs for bacteremia was 10%, whereas the LC-SF test allowed detection of DNA in 15% of cases. The LC-SF performance, based on its clinical relevance, was as follows: sensitivity, 78%; specificity, 99%; positive predictive value, 93%; and negative predictive value, 95%. Management was positively changed for four of eight (50%) of the patients because organisms were detected by the LC-SF test but not by BC. LC-SF results were quickly obtained compared to BC. Therefore, their results suggest that the LC-SF test may be a valuable complementary tool in the management of patients with clinically suspected sepsis [8]. Lehmann et al., 2008, by comparing multiplex real-time PCR identification results with conventional BC for 1,548 clinical isolates, reported an overall specificity of 98.8% for PCR; this specificity is significantly higher than the one of BC. This clearly shows how multiplex real-time PCR holds a promise for more rapid bacterial identification in clinical sepsis [17]. The same multiplex PCR was evaluated by Boyd and his coauthors on samples retrieved in the hospital and their results showed that, compared to BC, PCR is more sensitive to bacterial infections [21]. Tennant et al., 2015, have examined the sensitivity of q-PCR in endemic region of typhoid, and samples were taken from hospitals and healthy volunteers. In the field trials, the q-PCR diagnostic tool was 40% as sensitive as blood culture. However, when q-PCR positive specimens were considered to be true positives, blood culture only exhibited 28.57% sensitivity and a specificity of ≥90% for all comparisons. The q-PCR was significantly faster than blood culture in terms of detection of typhoid and paratyphoid infections [13].

All the 18 reviewed papers emphasize the need of using molecular techniques for the diagnosis of bBSIs. Warhurst et al., 2015, reported that SeptiFast real-time PCR is more rapid in the detection of BSIs though it has some limitations that must be handled over time [19]; this was emphasized by Wang et al., 2014, who mentioned that sepsis is one of the main causes of mortality due to therapy delay [22]. To overcome this challenge, molecular technique has to be used for rapid screening of bacteria. Tennant et al., 2015, investigated the use of gold standard method for diagnosis of enteric fever caused by* Salmonella typhi* or* Salmonella paratyphi* A or B in bone marrow culture [13]. However, because bone marrow aspiration is highly invasive, many hospitals and large health centers perform blood culture instead. Among other molecular techniques tried out, q-PCR was chosen with an increased sensitivity and specificity. Liesenfeld et al., 2014, worked on sepsis and considered a race to the death between the pathogens and the host immune system. In order to increase the speed of diagnosis, to improve sensitivity and the clinical benefit of detection of pathogens in the blood, molecular detection techniques for bacterial DNA have been implemented but are not very useful in each clinical use [12]. Lehmann et al., 2008, revealed that early detection of BSI is important in the clinical institution. Molecular diagnostic tools can contribute to a more rapid diagnosis in septic patients than BC. Here, multiplex real-time PCR-based assay for rapid detection of 25 clinically important pathogens directly from whole blood in less than 6 hours is presented [17]. Lecuit and Eloit, 2014, reported that gold standard technique suffers a number of limitations, including the need for a dedicated specialized staff and its intrinsic inefficiency to detect propagated fastidious bacteria such as* Treponema pallidum *and* Mycobacterium leprae*. BC has been progressively complemented and sometimes replaced by nucleic acid-based tests like PCR or Nucleic Acid Sequence Based Amplification (NASBA). The advantages of PCR are numerous: speed, low cost, automation, sensitivity, and specificity [16]. Jordana-Lluch et al., 2015, remarked that rapid identification of the etiological agent in BSI is of vital importance for the early administration of the most appropriate antibiotic therapy; thus, molecular methods may offer an advantage to current culture-based microbiological diagnosis [14]. The rapid administration of the most appropriate antimicrobial treatment is of interest for the survival of septic patients; therefore, a rapid method that enables direct diagnosis from analysis of a blood sample without culture is needed. A recently developed platform that couples broad-range PCR amplification of pathogen DNA with electrospray ionization mass spectrometry (PCR/ESI-MS) identifies any microorganism that might be present in whole clinical blood specimens [20]. The PCR/ESI-MS assay presents an advantage over the matrix-assisted laser desorption/ionization time-of-flight (MALDI-TOF) mass assay as it has been optimized to achieve a rapid diagnosis from direct clinical blood specimens. PCR/ESI-MS is a robust tool that offers an alternative for the diagnosis of BSI as it can be used alone and reliable results are provided following its new version that has been released recently [20]. The same molecular tool shows high specificity with a rate of positivity which is similar to that of BC; therefore, changes in its design would be needed to increase bacterial detection and to develop its automated version in clinical laboratories [15]. This will definitely lead to an improved sensitivity of PCR/ESI-MS.

Faria et al., 2015, indicated that each delay in antibiotic administration decreases the survival chance of the patient; then rapid diagnostic tools are needed and nucleic acid-based technologies and proteomic approaches are taking part in a more accurate diagnosis of bBSI [23]. The LC-SF test is the first DNA based test developed to detect microorganisms directly from blood sample without the need for prior incubation. Such a test has great potential to optimize the management of patients with suspected sepsis. In one paper, authors revealed that the rate of recovery from bacteremia was apparently better with the LC-SF and this has confirmed the ability of this test to improve the life of clinically ill patients [8]. LightCycler-SeptiFast (LC-SF), which is a real-time multiplex PCR test, can detect 25 common pathogens that cause BSI within few hours; as a matter of fact, LC-SF test can still provide valuable information for identifying the disease [18]. Carrara et al., 2013, reported that mortality from BSI is related to diagnostic delay and the use of empirical antibiotic therapy; and PCR-based diagnostic assays decrease empirical treatment and improve patient outcome [15]. According to Bacconi et al., 2014, developing a more automated, rapid, and sensitive molecular tool capable of detecting the diverse agents of bBSI at low titers has been challenging but would contribute enormously to the reduction of inappropriate treatment [6].

### 3.2. Discussion

Globally, bBSIs are the most common cause of sepsis and characterized by high mortality rates [24]. Incidence of bBSIs is still high in developing countries; for instance, in Africa, bBSIs have been reported among 10.7% of the children and among 13.9% of the adult patients with severe febrile illness admitted to hospitals [25]. Rapid, accurate diagnosis and treatment of bBSIs are crucial for the survival of the patient. Kumar et al. reported a strong relationship between delay in appropriate antibiotic treatment and survival of patients with severe bacteremia [26]. Correct treatment within the first hour was reported to be associated with a survival rate of 79.9% and each hour of delay associated with an average decrease in survival of 7.6% [27]. The spread of antibiotic resistant bacteria is considered to be one of the most important threats to the global public health.

In most settings, diagnosis of bBSIs is still based on conventional blood culture followed by the identification and antibiotic susceptibility testing of the grown bacteria. However, blood culture shows a sensitivity rate of only 60% and is not only time-consuming, but also laborious. In addition, it possesses serious biosafety risks since the bacteria are grown in vitro for subsequent microbiological analysis. In the last decades, there have been improvements in enriched growth media towards automated blood culture systems such as Bactec and BacT/Alert. This automated system uses software allowing a quicker detection of grown bacteria in culture; and this has significantly decreased contamination rates [28, 29]. In spite of this automated BC system, the technique still remains slow (up to 3 days) and not sensitive enough for accurate diagnosis of bBSIs. Molecular diagnostic methods are an interesting alternative to BC since they lead to a sensitive, specific, and rapid (<3 hours) detection of the bacterial genetic materials in blood samples [12]. Most molecular diagnostic tools are based on the polymerase chain reaction (PCR). This technique amplifies a specific region in the bacterial genome to levels sufficient for detection. However, these molecular tools have not been implemented in clinical settings of developing countries yet because they require specific laboratory facilities and skills [12].

In this review, it is clearly noticed that PCR-based techniques increase the sensitivity and specificity in the detection of bBSI. In addition, the use of such molecular techniques in diagnosing bBSI has reduced associated risks such as long turnaround time and false negative and positive results and has contributed to easy identification of fastidious bacteria and prevention of empirical therapy. All reviewed papers emphasized more the effectiveness and rapidity of molecular techniques. Reviewed techniques are mainly based on automated DNA extraction, PCR set-up, PCR amplification, amplicons purification, and PCR/ESI-MS. They overall lead to microbial identification from whole blood in not more than 6 hours [14]. Among other promising molecular tools, fluorescent in situ hybridization (FISH) has also the ability to detect pathogen within a shorter time, 2 to 3 hours [12].

Perfect diagnostic technology is able to identify the infecting organism and also the determinants of antibiotic resistance in a timely fashion so that the administration of appropriate therapy could start soon after diagnostic results. The ideal molecular method would analyze a patient's blood sample and provide all the information needed to immediately direct the optimal antimicrobial therapy for bBSI [30]. Therefore, the potential of molecular tools such as real-time PCR technology is to address this problem based on their ability to detect minute amounts of pathogenic DNA in patient blood samples and generate results in less than 6 hours of the test.

From a theoretical point of view, PCR-based diagnostic techniques hold promise for sensitive and specific detection of target pathogen within a short time. In contrast, for a good and accurate identification of a pathogen in bBSI, several parallel or serial specific PCR analyses or a more universal PCR assay followed by specific probe hybridization or sequencing of the targeted bacteria would bring more promise [17]. The rapid detection of pathogens in blood of septic patients is essential for adequate antimicrobial therapy and exact knowledge about causative microbial agent. For instance, when it comes to targeting bacteremia, the accuracy of the LC-SF is high enough to reach 80% and its specificity reaches 95%. This has also revealed the improved discrimination for the specific bacteremia outcome as compared to multiple target bacteremia [18]. Multiplex PCR has the potential to rapidly identify BSI, compensating for the loss of blood culture sensitivity. For instance, in all Italian hospitals, multiplex PCR (the LightCycler-SeptiFast, LC-SF, test) was compared to routine blood culture with samples obtained from 803 patients with suspected sepsis. In this study, excluding results attributable to contaminants, SeptiFast showed a sensitivity of 85.0% and a specificity of 93.5% compared to blood culture; and the rate of positive results was significantly higher with SeptiFast, 14.6%, than blood culture, 10.3% [12]. In a related study conducted in Pakistan, real-time PCR was significantly faster at detecting and identifying* Salmonella typhi* or* Salmonella paratyphi* A than classical microbiological techniques; though this technique is more sensitive it has missed some microbes detected by BC [13]. PCR of bacterial DNA seems to be the most sensitive molecular technique nowadays; even though more has to be done to improve its sensitivity, PCR stands to be the future direction tool in bBSI diagnosis.

Broad-range assays, with primers targeting variable regions in the16S rRNA or 18S/23S rRNA gene, present clinical applicability for diagnosis of bBSI due to their short turnaround time and ability to directly detect any noncultivable or cultivable pathogens in patients' blood sample [21]. In addition, other molecular techniques in comparison to PCR are emerging. For instance, Liesenfeld et al., 2014, described that fluorescent in situ hybridization (FISH) is among the available molecular techniques and has ability to detect pathogen within 2-3 h; another technique is based on chemiluminescent DNA probes (rRNA) and works like normal PCR assays [12]. Furthermore, another novel approach in molecular diagnosis of bBSIs, 16S metagenomics, has been recently developed [31]. 16S metagenomics consists in parallel sequencing of the bacterial 16S ribosomal RNA (rRNA) gene using next generation sequencing (NGS) technologies. The same technique has shown a superior sensitivity compared to standard blood culture during its proof-of-concept which was conducted in 75 children with severe febrile illness in Burkina Faso [31]. The use of microarrays and biomarkers has been also exploited and investigated for their inclusion in the diagnostic package of bBSI.

Current microarray-based techniques include Prove-It and Verigene tests. Prove-It consists in multiplex PCR in combination with microarray and can detect 60 bacterial pathogens in a positive culture sample [12]. It mainly detects two antibiotic resistant genes, mecA and vanA/B, with a total assay time of 3.5 hours [32]. Verigene, a bacterial nucleic acid-based microarray assay, can detect mecA and vanA/B resistant genes in addition to 13 gram-positive bacteria in a total assay time of 2.5 hours [12, 32].

Next to microarray-based tools, there is an emergence of various assays targeting biomarkers. Most of these techniques target endotoxins; acute-phase protein biomarkers such as C-reactive protein (CRP), lipopolysaccharide-binding protein (LBP), procalcitonin (PCT), pentraxin, serum amyloid A, ceruloplasmin, and alpha 1 acid glycoprotein; cytokines and chemokines; coagulation biomarkers; soluble receptor and cell surfaces [33]. The detection of endotoxins produced by gram-negative bacteria that might be circulating in the blood of septic patients was reported to be inhibited by other various products such as fungal cell wall components and plasma proteins [33, 34]. The CRP, usually released by liver upon inflammation during infection, has been exploited in sepsis diagnosis, especially when it comes to the assessment of the occurrence of bBSI [35]. Lipopolysaccharide-binding protein (LBP), as an acute-phase reactant binding to the lipopolysaccharide of gram-negative bacteria, levels increase during the acute-phase stage up to 200 *μ*g/mL [36]; thus, it is a good marker for the severity or outcome of the infection. However, this biomarker is not recommended for use in clinical settings as it failed to distinguish between gram-negative and gram-positive bacteremia [37]. The PCT is the mostly used protein marker in most of the parts of the world; it has the potential to distinguish between sepsis and system inflammatory respiratory syndrome (SIRS) and can determine the bacterial load or also guide the antibiotic therapy in ICU [33]. PCT is produced in response to bacterial endotoxin or immune mediators such as interleukin-1*β*, tumor necrosis factor-*α*, and interleukin-6 [38]. Pentraxin, a superfamily of immune system proteins, is still being investigated on its potential to differentiate among sepsis, septic shock, and SIRS [39]. For the rest of other acute-phase proteins, amyloid A, ceruloplasmin, alpha 1 acid glycoprotein, and hepcidin are reported to be elevated in septic patients [40]. The secretion of cytokines is simultaneously done in both proinflammatory and anti-inflammatory forms from the initial stage of infection; the rate of cytokines is higher in septic patients compared to nonseptic ones [40, 41]. However, cytokines present a limited usefulness as sepsis biomarkers because they can sometimes be linked to other noninfectious diseases as well [33]. Chemokines such as macrophage migration inhibitory factor (MIF) and high mobility-group box 1 provide value in the assessment of the immunological response. However, they also fail to distinguish between infectious and noninfectious systemic inflammation [42]. Among other markers, we can mention the triggering receptor expressed on myeloid cells 1 (sTREM-1), soluble urokinase-type plasminogen activator (suPAR), proadrenomedullin (proADM), and polymorphonuclear CD64 index. They are all promising markers for the diagnosis and prognosis of septic patients [43, 44]. However, they still need further investigation through larger studies. Overall, the use of combinatorial biomarkers could definitely lead to an improved diagnostic power and follow-up of septic patients.

Following the results of this systematic review, we believe that next generation molecular tools constitute a new and powerful approach that could identify main species causing bBSIs and detect their respective genetic markers responsible for antibiotic resistance. Molecular diagnostic tools would provide unique possibilities in the surveillance of bBSI. Surveillance studies at all health system levels are important to know the causative agents of bBSI and devise appropriate interventions to control the spread of antibiotic resistance and guide physicians in deciding which adequate antibiotics to prescribe.

## 4. Conclusion

In conclusion, it is noteworthy that molecular techniques are now emerging as another promising option for diagnosis of bBSI. In this review, PCR-based assays were highly reported to have significantly changed diagnostics of bBSI by increasing a bit sensitivity, specificity, and test accuracy overall. These techniques are generally good as they yield much better and reliable results in much shorter time than BC. In countries where trials have been conducted, reports have emphasized the accuracy of test results leading to a timely and right antibiotic administration. Although the cost of some of the newly developed techniques is still comparably high to be used in some poor endemic settings, we hope to get cheap, accurate, and fast methods requiring low training soon. This will be achieved through the advances in genomics, metagenomics, transcriptomics, metatranscriptomics, and proteomics together with much collaboration in international health services. Thus, the use of these sophisticated tools will soon shift from research settings and developed world to clinical settings and developing world. This will obviously tackle the challenge of usual delay in test results deliverance when using conventional BC. We are all convinced that elaboration of a quick and affordable tool for detecting bacterial pathogens in patients' blood sample is of great interest in global public health.

## Figures and Tables

**Table 1:** Summary of papers included in this review.

First author	Year of publication	Study period	Study country	Age range	Sample size	Patient's setting	Type of diagnostic tool	Design	Cases with bBSIs	Performance	Findings
Faria	2015	Not stated	Canada	Adult	Not stated	Hospital based	Illumina sequencing following PCR amplification of 16S rDNA	Lysis of cells followed by DNA extraction	Not stated	High but not specified	Molecular profiling is more accurate than blood culture
Jordana-Lluch	2014	2012-2013	Spain	Not stated	Not stated	Review type of study	PCR/ESI-MS	Use of amplicons	Not stated	Sensitivity 75%, specificity 92%	This molecular technique provides fast diagnosis of clinical samples
Venkatesh [9]	2010	Not stated	USA	Neonatal	Not stated	Hospital, ICU	Microarray and PCR	Detection of organism by hybridization and amplification	Not stated	Sensitivity of 98.7%, specificity of 99% for microarray, sensitivity and specificity of 86.4 and 99.0%, respectively, for PCR	These methods are more feasible compared to BC
Warhurst	2015	Not stated	UK	Not stated	Not stated	Review study	SeptiFast real-time PCR	Not stated	Not stated	60.8% sensitivity, 86.3% specificity	PCR-based assays are more specific but suffer from low sensitivity values
Carrara	2013	4/2011–9/2011	Spain	Not stated	267	Hospital, ICU	Multiplex PCR assay, the Magicplex Sepsis Test	Not stated	98	Sensitivity 65% (52–76%), specificity 92% (87–95%)	Can detect even fastidious bacteria but still needs some improvement to increase sensitivity
Ecker	2010	Not stated	USA	Both adult and children	Not stated	Not stated	PCR-ESI/MALDI-TOF/PCR-EIA	Sample extraction, lysis and enrichment with reagent	Not stated	Sensitivity, specificity, and positive and negative predictive values were 95.0, 98.8, 95.0, and 98.8%, respectively	They are more accurate, sensitive, and fast compared to blood culture
Boyd	2014	2011–2013	Canada	Not stated	245	Not stated	Multiplex PCR	Use of 16RNA for bacterial identification	Not stated	High specificity but low sensitivity	The use of molecular techniques will improve life in septic patients
Lecuit	2014	Not stated	France	Not stated	Not stated	Review study	Multiplex PCR	Bacterial typing is done by detecting conserved 16S rRNA regions	Not stated	95% sensitivity, 92% specificity	Further optimization of multiplex PCR is recommended
Reddy [10]	2010	Not stated	Tanzania, Malawi, and Kenya	Adults and infants	221	Hospital based	PCR	Not stated	136	Moderate	Ease of identifying bacterial etiologies
Lehmann	2008	Not stated	Germany	Not stated	574	Healthy volunteers	Multiplex real-time PCR	Compare PCR amplicons to the conserved regions	Not stated	Higher performance than blood culture	PCR-based techniques chosen for their sensitivity and specificity
Wallet	2010	Not stated	France	Not stated	Not stated	Hospital, ICU	LightCycler-SeptiFast (LC-SF)	Not stated	Not stated	Sensitive at 78%, specific at 99%	LC-SF is of valuable interest for patients with sepsis
Bacconi	2014	Not stated	Maryland	Not stated	331	Hospital samples	PCR/EI-MS	Cells are lysed and followed by DNA extraction by an automated instrument	35	83% sensitivity, 94% specificity	Rapid detection and identification of microbes
Tennant	2015	Not stated	Pakistan/Karachi	Adult	Not stated	Hospital samples	q-PCR	Not stated	Not stated	40% sensitivity, >90% specificity	Use of multiple methodologies to increase accuracy
Wang	2014	Not stated	Republic of Korea	Both adult and children	Not stated	Hospital, ICU	The real-time PCR TaqMan assay	DNA extraction from a colony of blood culture	Not sated	Sensitivity of 100% and specificity of 89.5%	Molecular techniques are more specific than BC
Chang	2013	2011–2013	USA	Adult	34	Review type of study	LC-SF, multiplex real-time PCR	Not stated	18	High specificity and moderate sensitivity	LC-SF multiplex real-time PCR gives more promising results than BC
Liesenfeld	2014	Not stated	USA	Not stated	Not sated	Review type of study	Emphasis on commercially available molecular techniques	Not stated	Not stated	The most reported one is the PCR of 84% sensitivity and 94% specificity	Molecular methods have advantages in microbial identification, but they must be refined in a good algorithm
Dark	2011	Not stated	UK	Adult	600	Hospital, ICU	Multiplex real-time PCR	BC followed by DNA extraction and PCR	Not stated	95% specific and 87% sensitive	PCR-based techniques are better than blood culture when it comes to assay time, sensitivity, and specificity values
Jordana-Lluch	2015	2012–2014	Spain	Not stated	410	Hospital based	Multiplex PCR	Automated DNA extraction followed by PCR	Not stated	Sensitivity of 76.9% and specificity of 87.2%	A promising technology to detect a wide range of bacterial microbes

## References

[1] Angus D. C., Van der Poll T. (2013). Severe sepsis and septic shock. *The New England Journal of Medicine*.

[2] Loonen A. J. M., Wolffs P. F. G., Bruggeman C. A., van den Brule A. J. C. (2014). Developments for improved diagnosis of bacterial bloodstream infections. *European Journal of Clinical Microbiology and Infectious Diseases*.

[3] Ståhl A.-L., Arvidsson I., Johansson K. E. (2015). A novel mechanism of bacterial toxin transfer within host blood cell-derived microvesicles. *PLoS Pathogens*.

[4] Fenollar F., Raoult D. (2007). Molecular diagnosis of bloodstream infections caused by non-cultivable bacteria. *International Journal of Antimicrobial Agents*.

[5] Schrenzel J. (2007). Clinical relevance of new diagnostic methods for bloodstream infections. *International Journal of Antimicrobial Agents*.

[6] Bacconi A., Richmond G. S., Baroldi M. A. (2014). Improved sensitivity for molecular detection of bacterial and candida infections in blood. *Journal of Clinical Microbiology*.

[7] Kumar A., Ellis P., Arabi Y. (2009). Initiation of inappropriate antimicrobial therapy results in a fivefold reduction of survival in human septic shock. *Chest*.

[8] Wallet F., Nseir S., Baumann L. (2010). Preliminary clinical study using a multiplex real-time PCR test for the detection of bacterial and fungal DNA directly in blood. *Clinical Microbiology and Infection*.

[9] Venkatesh M., Flores A., Luna R. A., Versalovic J. (2010). Molecular microbiological methods in the diagnosis of neonatal sepsis. *Expert Review of Anti-Infective Therapy*.

[10] Reddy E. A., Shaw A. V., Crump J. A. (2010). Community-acquired bloodstream infections in Africa: a systematic review and meta-analysis. *The Lancet Infectious Diseases*.

[11] Dark P., Dunn G., Chadwick P. (2011). The clinical diagnostic accuracy of rapid detection of healthcare- associated bloodstream infection in intensive care using multipathogen real-time PCR technology. *BMJ Open*.

[12] Liesenfeld O., Lehman L., Hunfeld K. P., Kost G. (2014). Molecular diagnosis of sepsis: new aspects and recent developments. *European Journal of Microbiology & Immunology*.

[13] Tennant S. M., Toema D., Qamar F. (2015). Detection of typhoidal and paratyphoidal salmonella in blood by real-time polymerase chain reaction. *Clinical Infectious Diseases*.

[14] Jordana-Lluch E., Giménez M., Quesada M. D. (2015). Evaluation of the broad-range PCR/ESI-MS technology in blood specimens for the molecular diagnosis of bloodstream infections. *PLoS ONE*.

[15] Carrara L., Navarro F., Turbau M. (2013). Molecular diagnosis of bloodstream infections with a new dual-priming oligonucleotide-based multiplex PCR assay. *Journal of Medical Microbiology*.

[16] Lecuit M., Eloit M. (2014). The diagnosis of infectious diseases by whole genome next generation sequencing: a new era is opening. *Frontiers in Cellular and Infection Microbiology*.

[17] Lehmann L. E., Hunfeld K.-P., Emrich T. (2008). A multiplex real-time PCR assay for rapid detection and differentiation of 25 bacterial and fungal pathogens from whole blood samples. *Medical Microbiology and Immunology*.

[18] Chang S.-S., Hsieh W.-H., Liu T.-S. (2013). Multiplex PCR system for rapid detection of pathogens in patients with presumed sepsis-a systemic review and meta-analysis. *PLoS ONE*.

[19] Warhurst G., Dunn G., Chadwick P. (2015). Rapid detection of health-care-associated bloodstream infection in critical care using multipathogen real-time polymerase chain reaction technology: a diagnostic accuracy study and systematic review. *Health Technology Assessment*.

[20] Jordana-Lluch E., Giménez M., Quesada M. D., Ausina V., Martró E. (2014). Improving the diagnosis of bloodstream infections: PCR coupled with mass spectrometry. *BioMed Research International*.

[21] Boyd J. H., Russell J. A., Fjell C. D. (2014). The meta-genome of sepsis: host genetics, pathogens and the acute immune response. *Journal of Innate Immunity*.

[22] Wang H.-Y., Kim S., Kim H. (2014). Real-time PCR taqman assay for rapid screening of bloodstream infection. *Annals of Clinical Microbiology and Antimicrobials*.

[23] Faria M. M. P., Conly J. M., Surette M. M. G. (2015). The development and application of a molecular community profiling strategy to identify polymicrobial bacterial DNA in the whole blood of septic patients. *BMC Microbiology*.

[24] Gaieski D. F., Edwards J. M., Kallan M. J., Carr B. G. (2013). Benchmarking the incidence and mortality of severe sepsis in the United States. *Critical Care Medicine*.

[25] Prasad N., Murdoch D. R., Reyburn H., Crump J. A. (2015). Etiology of severe febrile illness in low- and middle-income countries: a systematic review. *PLoS ONE*.

[26] Kumar A., Roberts D., Wood K. E. (2006). Duration of hypotension before initiation of effective antimicrobial therapy is the critical determinant of survival in human septic shock. *Critical Care Medicine*.

[27] Pavese P., Maillet M., Vitrat-Hincky V. (2014). Evaluation of an intervention to improve blood culture practices: a cluster randomised trial. *European Journal of Clinical Microbiology & Infectious Diseases*.

[28] Mancini N., Carletti S., Ghidoli N., Cichero P., Burioni R., Clementi M. (2010). The era of molecular and other non-culture-based methods in diagnosis of sepsis. *Clinical Microbiology Reviews*.

[29] Kim N.-H., Kim M., Lee S. (2011). Effect of routine sterile gloving on contamination rates in blood culture. *Annals of Internal Medicine*.

[30] Ecker D. J., Sampath R., Li H. (2010). New technology for rapid molecular diagnosis of bloodstream infections. *Expert Review of Molecular Diagnostics*.

[31] Decuypere S., Meehan C. J., Van Puyvelde S. (2016). Diagnosis of bacterial bloodstream infections: a 16S metagenomics approach. *PLOS Neglected Tropical Diseases*.

[32] Lebovitz E. E., Burbelo P. D. (2013). Commercial multiplex technologies for the microbiological diagnosis of sepsis. *Molecular Diagnosis and Therapy*.

[33] Reinhart K., Bauer M., Riedemann N. C., Hartog C. S. (2012). New approaches to sepsis: molecular diagnostics and biomarkers. *Clinical Microbiology Reviews*.

[34] Marshall J. C., Foster D., Vincent J.-L. (2004). Diagnostic and prognostic implications of endotoxemia in critical illness: results of the MEDIC study. *The Journal of Infectious Diseases*.

[35] Uzzan B., Cohen R., Nicolas P., Cucherat M., Perret G.-Y. (2006). Procalcitonin as a diagnostic test for sepsis in critically ill adults and after surgery or trauma: a systematic review and meta-analysis. *Critical Care Medicine*.

[36] Tobias P. S., Mathison J., Mintz D. (1992). Participation of lipopolysaccharide-binding protein in lipopolysaccharide-dependent macrophage activation. *American Journal of Respiratory Cell and Molecular Biology*.

[37] Sakr Y., Burgett U., Nacul F. E., Reinhart K., Brunkhorst F. (2008). Lipopolysaccharide binding protein in a surgical intensive care unit: a marker of sepsis?. *Critical Care Medicine*.

[38] Müller B., Becker K. L., Schächinger H. (2000). Calcitonin precursors are reliable markers of sepsis in a medical intensive care unit. *Critical Care Medicine*.

[39] Vänskä M., Koivula I., Hämäläinen S. (2011). High pentraxin 3 level predicts septic shock and bacteremia at the onset of febrile neutropenia after intensive chemotherapy of hematologic patients. *Haematologica*.

[40] Tamayo E., Fernández A., Almansa R. (2011). Pro- and anti-inflammatory responses are regulated simultaneously from the first moments of septic shock. *European Cytokine Network*.

[41] van Nieuwkoop C., Bonten T. N., van't Wout J. W. (2010). Procalcitonin reflects bacteremia and bacterial load in urosepsis syndrome: a prospective observational study. *Critical Care*.

[42] Karlsson S., Pettilä V., Tenhunen J., Laru-Sompa R., Hynninen M., Ruokonen E. (2008). HMGB1 as a predictor of organ dysfunction and outcome in patients with severe sepsis. *Intensive Care Medicine*.

[43] Donadello K., Scolletta S., Covajes C., Vincent J.-L. (2012). SuPAR as a prognostic biomarker in sepsis. *BMC Medicine*.

[44] Struck J., Tao C., Morgenthaler N. G., Bergmann A. (2004). Identification of an Adrenomedullin precursor fragment in plasma of sepsis patients. *Peptides*.

